# Exploring the Multifactorial Landscape of Penile Cancer: A Comprehensive Analysis of Risk Factors

**DOI:** 10.3390/diagnostics14161790

**Published:** 2024-08-16

**Authors:** Ugo Amicuzi, Marco Grillo, Marco Stizzo, Michelangelo Olivetta, Simone Tammaro, Luigi Napolitano, Pasquale Reccia, Luigi De Luca, Andrea Rubinacci, Giampiero Della Rosa, Arturo Lecce, Paola Coppola, Salvatore Papi, Francesco Trama, Lorenzo Romano, Carmine Sciorio, Lorenzo Spirito, Felice Crocetto, Celeste Manfredi, Francesco Del Giudice, Matteo Ferro, Bernardo Rocco, Octavian Sabin Tataru, Raffaele Balsamo, Giuseppe Lucarelli, Dario Del Biondo, Biagio Barone

**Affiliations:** 1 Division of Urology, Department of Surgical Sciences, AORN Sant’Anna e San Sebastiano, 81100 Caserta, Italy; ugo.amicuzi@aorncaserta.it; 2 Department of Urology, Ospedale del Mare, ASL NA1 Centro, 80147 Naples, Italy; grillomarco@inwind.it (M.G.); dario.delbiondo@aslnapoli1centro.it (D.D.B.); 3 Urology Unit, Department of Woman, Child and General and Specialized Surgery, University of Campania Luigi Vanvitelli, 80131 Naples, Italy; marco.stizzo@policliniconapoli.it (M.S.); lorenzospirito@msn.com (L.S.); manfredi.celeste@gmail.com (C.M.); 4 Urology Unit, Gaetano Fucito Hospital, AOU San Giovanni di Dio e Ruggi d’Aragona, 84085 Mercato San Severino, Italy; m.olivetta@sangiovannieruggi.it; 5 Department of Neurosciences and Reproductive Sciences and Odontostomatology, University of Naples Federico II, 80131 Naples, Italy; simone.tammaro95@gmail.com (S.T.); luigi.napolitano@unina.it (L.N.); andrearubinacci1@gmail.com (A.R.); giampierodellarosa@gmail.com (G.D.R.); arturo.lecce92@gmail.com (A.L.); coppola997@gmail.com (P.C.); salvatorepapi@blu.it (S.P.); lorenzo.romano@unina.it (L.R.); felice.crocetto@unina.it (F.C.); 6 Urology Unit, AORN Ospedali dei Colli, Monaldi Hospital, 80131 Naples, Italy; pasquale.reccia@ospedalideicolli.it (P.R.); raffaele.balsamo@ospedalideicolli.it (R.B.); 7 Division of Urology, Department of Surgical Multispecialty, AORN Antonio Cardarelli, 80131 Naples, Italy; luigideluca86@gmail.com; 8 Urology Complex Unit, ASL Napoli 2 Nord ‘Santa Maria delle Grazie’ Hospital, 80078 Pozzuoli, Italy; francescotrama@gmail.com; 9 Urology Unit, ASST Ospedale Manzoni, 23900 Lecco, Italy; carmine.sciorio@gmail.com; 10 Department of Urology, University Sapienza, 00185 Rome, Italy; francesco.delgiudice@uniroma1.it; 11 2nd Unit of Urology, Department of Health Science, University of Milan, ASST Santi Paolo and Carlo, Via A. Di Rudini 8, 20142 Milan, Italybernardo.rocco@gmail.com (B.R.); 12 Department of Simulation Applied in Medicine, The Institution Organizing University Doctoral Studies (I.O.S.U.D.), George Emil Palade University of Medicine, Pharmacy, Sciences, and Technology from Târgu Mureș, 540142 Târgu Mureș, Romania; sabin.tataru@umfst.ro; 13 Urology, Andrology and Kidney Transplantation Unit, Department of Emergency and Organ Transplantation, University of Bari, 70124 Bari, Italy; giuseppe.lucarelli@uniba.it; 14 Department of Urology, Ospedale San Paolo, ASL NA1 Centro, 80125 Naples, Italy

**Keywords:** penile cancer, risk factors, epidemiology, human papillomavirus, circumcision, early detection

## Abstract

Penile cancer, while rare, is a critical public health issue due to its profound impact on patients and the complexities of its management. The disease’s multifactorial etiology includes risk factors such as HPV infection, poor hygiene, smoking, genetic predispositions, and socioeconomic determinants. This article provides a comprehensive review and analysis of these diverse risk factors, aiming to enhance understanding of the disease’s underlying causes. By elucidating these factors, the article seeks to inform and improve prevention strategies, early detection methods, and therapeutic interventions. A nuanced grasp of the multifactorial nature of penile cancer can enable healthcare professionals to develop more effective approaches to reducing incidence rates and improving patient outcomes.

## 1. Introduction

**Figure 1:**
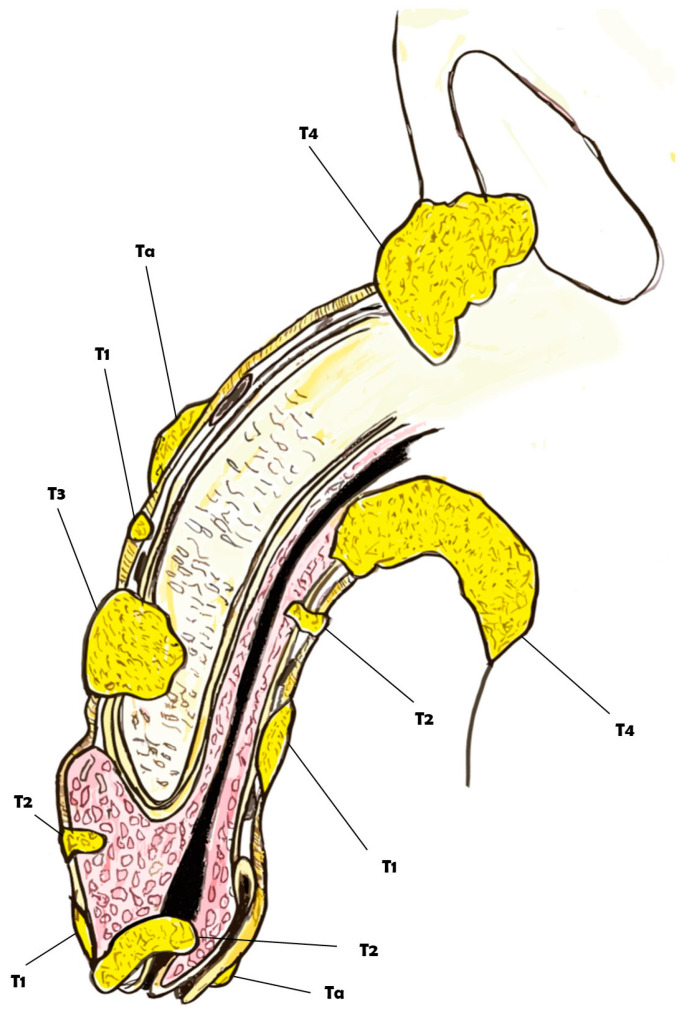
Overview of the clinical classification of the TNM for penile cancer.

**Figure 2:**
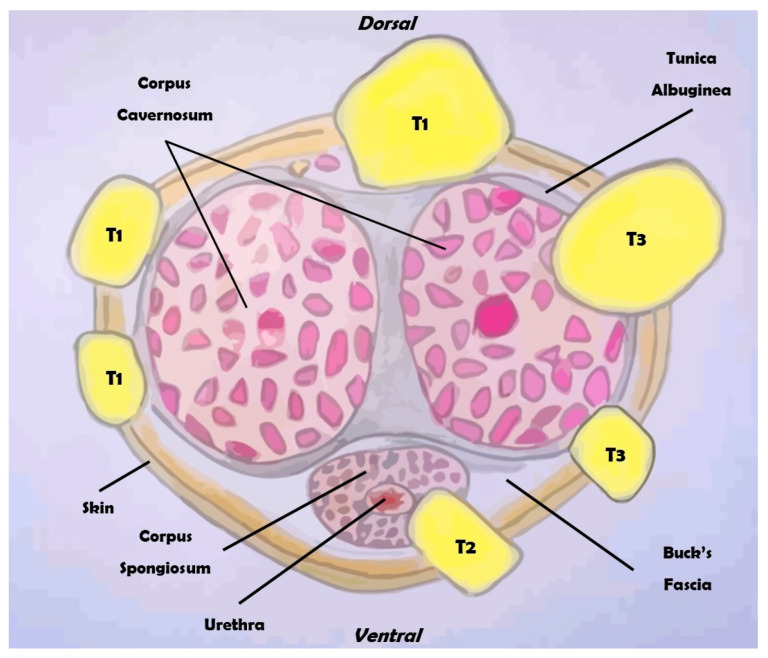
Overview of the clinical classification of the TNM for penile cancer (section).

Penile cancer, although an extremely rare malignancy, currently represents a substantial global health problem. In fact, with an overall incidence of 1:100,000 in Europe, penile cancer is characterized by a high mortality rate and is also associated with a significant impact on quality of life [1,2]. Patients diagnosed with this condition not only face the psychological and emotional stress common to cancer diagnoses but also endure the specific distress of a disease that affects intimate parts of the body [3]. The treatments for penile cancer are often highly mutilating, involving surgical procedures that can profoundly alter the patient’s body image and sense of masculinity. Such treatments frequently lead to the loss of a key aspect of male identity, triggering severe anxiety and depression. This psychological burden can have a detrimental effect on various aspects of life, particularly in the sexual sphere and everyday interpersonal relationships. The stigma and physical changes associated with the treatment can lead to feelings of inadequacy and isolation, exacerbating the mental health challenges faced by these patients [4,5]. Understanding the multifactorial nature of penile cancer is crucial for developing effective strategies for prevention, early detection, and treatment. Numerous risk factors contribute to the development of penile cancer, including HPV infection, poor hygiene, smoking, and other lifestyle factors. Genetic predisposition and socioeconomic status also play significant roles in the incidence and progression of this disease. By comprehensively reviewing and analyzing these diverse risk factors, we can gain deeper insights into the etiology of penile cancer and identify potential avenues for intervention [6,7,8]. Accurate staging of penile cancer is essential for determining the appropriate treatment and predicting outcomes. The TNM classification system, which assesses tumor size, lymph node involvement, and metastasis, is commonly used for this purpose (Figure 1 and Figure 2).

This article aims to provide a thorough examination of the risk factors associated with penile cancer, and, in particular, penile squamous cell carcinoma (SCC), the most common malignancy involving the penile region. By shedding light on its multifactorial nature, the goal is to enable the development of more effective prevention strategies and therapeutic interventions. Unless otherwise specified, all risk factors discussed pertain specifically to penile SCC. Through enhanced awareness and targeted efforts, it is possible to reduce the incidence and improve the outcomes for patients suffering from this challenging and often devastating condition.

## 2. Global Burden of Penile Cancer: Epidemiology and Trends

Penile cancer is a rare disease for which only 26,000 cases are estimated globally per year [9]. Globally, the incidence and prevalence of penile cancer exhibit notable disparities across regions [10]. According to recent data from the World Health Organization (WHO), the highest incidence rates are observed in parts of South America, Africa, and Southeast Asia; in fact, penile cancer can constitute up to 10% of male malignancies in these areas [11]. Conversely, developed regions such as North America and Western Europe report lower incidence rates [12]. These geographical variations are believed to be influenced by a combination of genetic, environmental, and lifestyle factors, which form the focus of our investigation [13]. The prevalence of penile cancer is often under-reported due to social stigma, limited healthcare access, and disparities in healthcare infrastructure [14]. Despite its relative rarity compared to other cancers, the impact on affected individuals and their quality of life is substantial [15]. The precise tumorigenesis of penile cancer is still poorly understood, but several risk factors have been identified. As we delve into the multifactorial landscape of penile cancer’s risk factors, it becomes apparent that genetic predisposition, human papillomavirus (HPV) infection, hygiene practices, lifestyle factors, and socioeconomic determinants collectively contribute to the complex etiology of this disease [6,14,16]. Several studies have provided compelling evidence supporting the existence of a genetic component in penile cancer susceptibility [17,18,19,20]. Familial aggregation of cases, where individuals with a family history of penile cancer are at a higher risk, suggests potential hereditary influence and/or shared environmental influences [21,22,23]. These findings underscore the importance of genetic investigations to identify specific risk alleles and understand the pathways through which they contribute to the development of penile cancer. The rarity of penile cancer has posed challenges in conducting large-scale genetic studies. However, recent advances in genomics and collaborative efforts among research institutions have facilitated the identification of potential genetic markers associated with penile cancer risk [18]. Genome-wide association studies (GWASs) and next-generation sequencing technologies have become invaluable tools in unraveling the genetic underpinnings of this disease. The impact of socioeconomic factors on penile cancer incidence unveils a multifaceted and intricate landscape where access to healthcare, educational opportunities, and cultural practices intertwine to shape the burden of this rare malignancy. In-depth scrutiny of these socioeconomic determinants illuminates profound disparities in disease occurrence among diverse strata of society, underscoring the imperative for tailored interventions to rectify these health inequities [24].

## 3. Etiology and Histopathology

**Figure 3:**
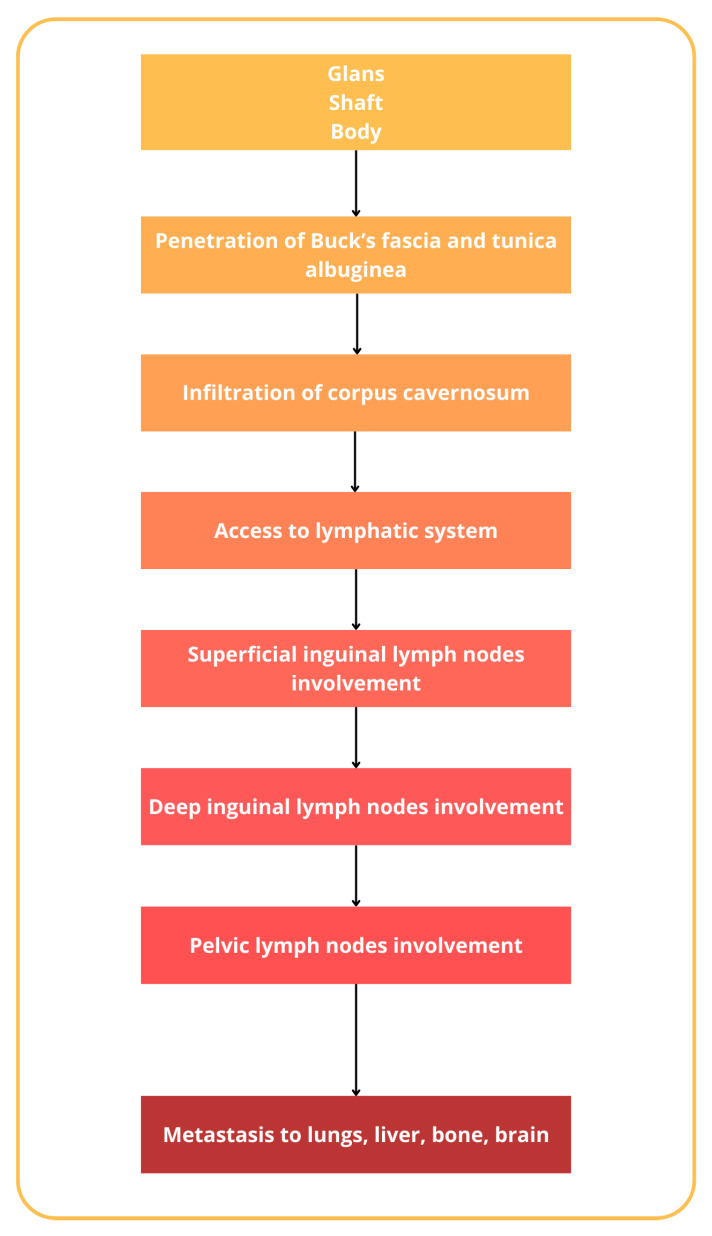
Natural history of penile cancer.

The incidence of penile cancer varies and is related to different factors, beginning, traditionally, as a painless lesion, nodules, lumps, or also ulcers on the glans penis or prepuce, with a great variation in appearance [25]. Regardless, penile cancer could originate from various cell types within the penis. The majority of penile cancers, around 95%, arise from the epithelial cells of the skin covering the penis, known as squamous cells. Less commonly, other types of penile cancer can develop from different cell types, such as melanocytes, fibroblasts, and blood vessel cells [26]. Squamous cell carcinoma could be further subdivided into the usual type (45–65%), papillary carcinoma (2–15%), warty condylomatous tumor (7–10%), basaloid carcinoma (4–10%), verrucous carcinoma (3–7%), and sarcomatoid (spindle cell) carcinoma (1–6%) [27,28]. The histopathological classification of penile cancer, as outlined by the World Health Organization (WHO), is reported in Table 1 [28,29,30]. The natural history of penile cancer includes several steps, beginning with the aforementioned lesions on the glans and prepuce, mostly, that gradually extend to involve the entire glans or the shaft of the penis [31]. It has to also be noted that in many older patients, phimosis could conceal the lesion and allow the silent progression of the lesion [32]. Eventually, the tumor erodes through the prepuce up to Buck’s fascia [33]. Penetration of Buck’s fascia and the tunica albuginea then permits the penetration to the corpus cavernosum and the invasion of the lymphatic system [34,35]. Penile cancer has a particular tendency for lymphatic spread to superficial and deep inguinal lymph nodes and, successively, pelvic lymph nodes [36,37]. Inguinal metastases gradually enlarge and ulcerate, producing complications related to uncontrollable locoregional growth. Distant metastases to the lungs, liver, bone, and brain are uncommon and usually occur in the late course of the disease [38,39] (Figure 3).

## 4. Risk Factors

### 4.1. Socioeconomic Disparities

Socioeconomic disparities are associated with increased cancer incidence in general; however, the relationship with penile cancer is not widely investigated in the literature [40,41,42]. One of the pivotal contributors to the socioeconomic gradient in penile cancer incidence is the variable access to healthcare resources. Disparities in healthcare infrastructure, both within and across nations, lead to differential access to preventive services, diagnostic capabilities, and timely medical interventions [43]. Individuals occupying lower socioeconomic strata often grapple with systemic barriers, resulting in delayed diagnoses, advanced disease stages upon presentation, and suboptimal treatment outcomes. The elucidation of these disparities highlights the urgency of dismantling barriers to healthcare access for vulnerable populations [44]. Another factor influencing the incidence and outcomes of penile cancer is associated with educational disparities. Education, indeed, as a key determinant of socioeconomic status, intricately influences penile cancer incidence [45,46]. Lower educational attainment has a profound impact on cancer risk, emphasizing how limited access to educational opportunities can correlate with a heightened vulnerability to risk factors. Individuals with lower educational backgrounds may face challenges in health literacy, inhibiting their understanding of the significance of proper hygiene practices, the role of lifestyle choices, and the importance of routine health check-ups [47,48]. Bridging the educational gap is essential for fostering awareness and empowering individuals to adopt preventive measures effectively [40]. A Swedish study reported an increased risk of invasive penile cancer in those with low disposable income and low education levels, but not with in situ disease. Moreover, a low educational level was associated with more advanced primary tumor stages [24]. In contrast, a Finnish study investigated the relationship between social class and genital cancer, including penile cancer, and found very little difference in tumor incidence between social classes. This discrepancy may be due to the Finnish study not capturing relevant measures of the socioeconomic gradient and risk factors related to the disease [45]. Geographical disparities, closely aligned with socioeconomic factors, add geographic specificity to the study of penile cancer. Certain regions, marked by limited healthcare infrastructure and socioeconomic challenges, may exhibit higher incidences of penile cancer. Developing countries show disparities in outcomes due to several factors, including lack of primary care services, low educational level, misdiagnosis, advanced tumor stage at diagnosis, follow-up due to labor circumstances, and delayed referral due to lack of specialized services in marginalized communities [49,50]. A retrospective Mexican study investigated geographical factors predisposing to a negative surgical outcome and higher mortality rate in patients with penile cancer. Interestingly, patients without a favorable surgical outcome (radical penectomy) were more likely to have been referred from a provincial hospital and to not have access to a primary care center [43]. Closely related, economic factors, often synonymous with socioeconomic status, play a pivotal role in shaping the penile cancer landscape [51]. Economic strain can exacerbate healthcare disparities, as uninsured or underinsured individuals may grapple with financial barriers to routine medical screenings and preventive measures [24,52]. McIntyre et al. analyzed data from referral centers in the southeastern United States, and found that patients with penile cancer more commonly lack health insurance. Additionally, patients who are heavy alcohol users or are uninsured present with advanced disease. These factors contribute to poorer prognosis in these patients. Furthermore, the economic burden of penile cancer treatment adds an additional layer of vulnerability, potentially leading to disparate health outcomes based on financial resources [53]. Understanding the economic dimensions of disease risk is fundamental for devising interventions that address financial disparities and ensure equitable access to healthcare services. Lastly, cultural practices and marital status introduce another layer of complexity to the socioeconomic disparities in penile cancer incidence. Certain populations may adhere to cultural traditions that inadvertently contribute to heightened risk factors, such as reluctance to seek medical attention for genital concerns or adherence to practices that foster chronic inflammation [13,54]. Conversely, marital status plays a role, with a population-based study showing decreased incidence rates in married men compared to those who are single. Men who are not currently married and men who live alone are at increased risk of penile cancer, just as a greater number of prior cohabitations, presumably a surrogate measure of relationship instability, is associated with increased risk of penile cancer [55].

### 4.2. Human Papillomavirus (HPV)

Human papillomavirus (HPV) has emerged as a pivotal contributor to penile cancer. HPV, a DNA virus belonging to the Papillomaviridae family, has been recognized as a causative agent for a spectrum of cancers, including cervical, anal, and penile cancers [56,57]. HPV subtypes are categorized based on their oncogenic potential, with high-risk types such as HPV-16 and HPV-18 posing a substantial risk for cancer development [58,59,60]. Penile cancer is often preceded by persistent HPV infection, primarily localized to the genital epithelium. The virus enters through microabrasions in the epithelial barrier, where it establishes an infection in basal keratinocytes. The outcome of this infection, ranging from clearance to persistent infection, is influenced by the complex interplay between viral and host factors—a critical determinant in the development of HPV-associated cancers. HPV-induced carcinogenesis is primarily driven by viral oncoproteins, namely E6 and E7 [61]. These proteins exhibit multifaceted activities that subvert cellular regulatory mechanisms, leading to uncontrolled cell growth and evasion of immune surveillance. The E6 oncoprotein disrupts the cell cycle and promotes genomic instability by binding to and facilitating the degradation of the tumor suppressor protein p53, which is crucial for cell cycle arrest and apoptosis [62]. Without functional p53, cells are more prone to genetic mutations and malignant progression. Conversely, the E7 oncoprotein targets the retinoblastoma protein (pRb) and other pocket proteins involved in cell cycle regulation [63]. By inactivating pRb, E7 facilitates the release of E2F transcription factors, promoting cell cycle progression [64]. The dysregulation of the cell cycle by E7 contributes to uncontrolled cell proliferation, a hallmark of cancer. The interplay between E6 and E7 is central to HPV-associated carcinogenesis, including penile cancer. Persistent HPV infection induces a spectrum of genetic and epigenetic alterations in infected cells, further propelling the transition to malignancy. Genomic instability, chromosomal aberrations, and mutations in key cellular genes contribute to the acquisition of cancerous traits [65,66,67,68,69]. DNA methylation and histone modifications induced by HPV alter the regulatory landscape of the infected cells, contributing to the silencing of tumor suppressor genes and activation of oncogenes. These genetic and epigenetic alterations induced by HPV play a synergistic role in driving the malignant transformation of infected cells [69]. HPV has evolved sophisticated mechanisms to evade host immune surveillance, allowing it to persist in the infected tissue and contribute to the development of cancer [70,71]. The virus modulates the host immune response through various strategies, including the inhibition of interferon production, interference with antigen presentation, and evasion of natural killer cell-mediated cytotoxicity. In the context of penile cancer, the ability of HPV to evade immune detection contributes to the establishment of persistent infection and the evasion of antitumor immune responses. Indeed, epidemiological studies have consistently demonstrated the association between high-risk HPV infection and the risk of penile cancer [68,72]. The prevalence of HPV in penile cancer specimens varies globally, with higher rates observed in regions where penile cancer incidence is elevated. Clinical implications of HPV in penile cancer extend beyond its role as a risk factor. HPV status has prognostic significance, with HPV-positive tumors often exhibiting distinct clinical and pathological characteristics. Studies suggest that HPV-positive penile cancers may have a more favorable prognosis compared to their HPV-negative counterparts [73,74,75]. Additionally, the link between HPV and penile cancer has implications for prevention strategies. HPV vaccination, initially designed to prevent cervical cancer, has shown promise in reducing the incidence of HPV-associated cancers, including penile cancer [67,76]. Integrating HPV vaccination into public health initiatives may serve as a preventive measure, particularly in populations with high HPV prevalence.

### 4.3. Smoking and Lifestyle Factors

Smoking, a well-established risk factor for different cancers, has been implicated in the etiology of penile cancer as a direct and independent dose-related risk factor [77]. Tsen et al., in their case–control study, showed a 2.4-fold risk increase in those who have ever smoked and an even higher incidence (OR 3.1) in current smokers [78]. The combustion of tobacco releases a complex mixture of carcinogenic compounds, including polycyclic aromatic hydrocarbons (PAHs), nitrosamines, and heavy metals. Considerable evidence indicates that in human cancers caused by cigarette smoking, PAHs, *N*-nitrosamines, aromatic amines, and certain volatile organic agents play a major role. The ability of a chemical to bind to DNA, either directly or after metabolic activation, is taken as evidence of mutagenic and carcinogenic potential. The group of compounds with well-established genotoxicity are polycyclic aromatic hydrocarbons (PAHs) [79]. These compounds, upon exposure, can exert direct genotoxic effects on the penile epithelium, promoting the initiation and progression of malignant transformation. They are known to bind to DNA, forming adducts that can lead to mutations in critical tumor suppressor genes and oncogenes. The activation of procarcinogens within tobacco smoke represents a mechanistic link between smoking and the genetic alterations observed in penile cancer. Understanding these molecular intricacies is pivotal for delineating the causal relationship between smoking and the pathogenesis of penile cancer [80]. Chronic smoking is associated with a persistent state of inflammation, and this inflammatory microenvironment can contribute to the development of penile cancer. Inflammation has been recognized as a hallmark of cancer, fostering a milieu that supports cell proliferation, angiogenesis, and tissue remodeling [81]. Cigarette smoke contains inflammatory mediators such as cytokines, chemokines, and reactive oxygen species (ROS) that can activate signaling pathways linked to carcinogenesis. These signaling cascades may contribute to the sustained inflammation observed in chronic smokers, creating an environment conducive to the progression of pre-malignant lesions to invasive penile cancer. Moreover, chronic inflammation has been implicated in immune evasion, enabling transformed cells to escape surveillance mechanisms and establish a foothold in the penile tissue [82]. The intricate interplay between smoking-induced inflammation and the immune response sheds light on the multifaceted impact of smoking on the carcinogenic process. The relationship between smoking and penile cancer is further nuanced by interactions with other lifestyle factors. Diet, obesity, sexual practices, and comorbid conditions collectively contribute to the intricate tapestry of penile cancer’s etiology. Dietary choices and obesity, often linked to lifestyle, can influence the risk of penile cancer. High-fat diets, low in fruits and vegetables, have been associated with an increased risk, potentially through mechanisms involving chronic inflammation, oxidative stress, and altered hormonal profiles [83]. Obesity, characterized by chronic low-grade inflammation and hormonal imbalances, may also contribute to the promotion of malignant transformation in penile tissue [84]. The interplay between smoking, diet, and obesity underscores the importance of adopting a holistic approach to lifestyle modifications for penile cancer prevention. Sexual practices, particularly those associated with an increased risk of sexually transmitted infections (STIs), play a role in penile cancer risk [85,86]. Smoking may exacerbate this risk by compromising the immune response and creating an environment conducive to persistent infections. Additionally, poor genital hygiene practices, often linked to lifestyle choices, may contribute to chronic inflammation and increase susceptibility to infections, further intertwining with the impact of smoking on penile cancer risk. The presence of comorbid conditions, such as diabetes and chronic inflammatory disorders, can accentuate the impact of smoking on penile cancer risk. Smoking-induced vascular damage and systemic inflammation may exacerbate the complications associated with comorbid conditions, creating a synergistic effect that heightens the susceptibility to penile cancer. Epidemiological studies have consistently demonstrated an association between smoking and an increased risk of penile cancer. The risk appears to be dose-dependent, with heavier and prolonged smoking linked to higher incidences of the disease [78]. The geographical and socioeconomic disparities observed in penile cancer incidence are mirrored in smoking patterns, further highlighting the need for a comprehensive understanding of lifestyle factors and their impact on cancer risk. The intricate relationship between smoking, lifestyle factors, and penile cancer underscores the potential for preventive interventions. Smoking cessation initiatives, coupled with lifestyle modifications (healthy diet, maintaining a normal body weight, and practicing safe sexual behaviors) represent avenues for reducing the risk of penile cancer. Public health campaigns aimed at raising awareness about the multifaceted nature of penile cancer risk factors can contribute to lifestyle changes at the individual and community levels [87]. Integrating smoking cessation programs with broader health promotion efforts may yield synergistic benefits, not only for penile cancer prevention but also for overall health and well-being. Lastly, although the exact mechanism is not fully understood, penile trauma is also considered a potential risk factor for the development of penile cancer. Chronic inflammation and scarring resulting from repeated trauma may contribute to potential carcinogenesis [88,89,90]. Further research is, however, needed to elucidate the relationship between trauma and penile cancer risk.

### 4.4. Phimosis and Hygiene Practices

Phimosis, a condition marked by the inability to retract the foreskin, emerges as a notable risk factor for penile cancer, drawing attention to the intricate interplay between anatomical factors and cancer susceptibility. The inability to retract the foreskin may lead to the accumulation of smegma, a mixture of exfoliated skin cells, bodily fluids, and microorganisms, creating a milieu conducive to inflammation and potential carcinogenesis [91]. Poor hygiene practices exacerbate this environment, fostering chronic irritation and inflammation that may contribute to the initiation and progression of disease [92,93]. Therefore, maintaining proper genital hygiene emerges as a crucial modifiable lifestyle factor in the development of penile cancer [94]. The discussion extends to the role of circumcision, a surgical intervention that has demonstrated a protective effect against penile cancer. While it is widely recognized as an effective preventive measure, especially when performed during the neonatal period, it does not eliminate the risk of the disease entirely [54,95]. This protective effect is attributed to several factors: circumcision reduces smegma accumulation, improves hygiene, decreases the risk of HPV and HIV transmission, and minimizes chronic inflammation and balanitis [96].

### 4.5. Chronic Inflammatory Conditions

Lichen sclerosus, a chronic inflammatory skin condition primarily affecting the genital area, has emerged as a significant risk factor for penile cancer, adding layers of complexity to our understanding of the intricate interplay between dermatological disorders and malignancies [27]. Lichen sclerosus is characterized by distinctive skin changes, manifesting as white, atrophic plaques with a predilection for the anogenital region. While the exact etiology of lichen sclerosus remains elusive, autoimmune factors, genetic predisposition, and hormonal imbalances are believed to contribute to its development. This dermatological condition predominantly affects females, although it can also affect males and, less commonly, children [97]. The chronic nature of lichen sclerosus underscores its potential to exert long-term effects on the affected tissue, leading to concerns regarding its association with an increased risk of malignancies, particularly penile cancer. Understanding the link between lichen sclerosus and penile cancer requires a thorough examination of the pathological processes at play. Chronic inflammation, a hallmark of lichen sclerosus, is a key player in the transformation of normal tissue into a pre-malignant or malignant state [98]. The persistent inflammatory microenvironment contributes to genetic and epigenetic alterations, disrupting cellular homeostasis and potentially paving the way for neoplastic progression [99]. The association between chronic inflammation and cancer is well established, and lichen sclerosus exemplifies this paradigm in the context of penile cancer. In addition to the common characteristic white plaques, patients may experience itching, pain, and discomfort, further complicating the clinical picture. The potential for misdiagnosis or delayed diagnosis is particularly concerning given the associated risk of penile cancer [100]. Vigilant monitoring of individuals with lichen sclerosus is paramount, emphasizing the need for regular clinical examinations and, in some cases, biopsies to assess for any malignant transformation. The epidemiological link between lichen sclerosus and penile cancer has been substantiated by numerous studies. Men with lichen sclerosus have been reported to face a significantly higher risk of developing penile cancer compared to the general population [101]. This increased risk prompts a critical examination of the underlying mechanisms that drive carcinogenesis in the context of lichen sclerosus. Beyond the scope of genetic and epigenetic alterations induced by chronic inflammation, the potential role of the altered microenvironment in supporting cancer progression warrants in-depth investigation [102]. Furthermore, the association between lichen sclerosus and penile cancer raises questions about potential biomarkers that could aid in risk stratification and early detection. Identifying specific molecular markers associated with the transition from lichen sclerosus to penile cancer could offer valuable insights into the disease’s natural history and facilitate the development of targeted screening strategies [103]. The integration of genomics, transcriptomics, and proteomics in the study of lichen sclerosus-associated penile cancer may uncover novel molecular signatures that inform both prognosis and therapeutic approaches. The implications of lichen sclerosus as a risk factor for penile cancer extend beyond the realms of diagnosis and molecular understanding. The recognition of this association emphasizes the importance of a multidisciplinary approach to patient care, involving dermatologists, urologists, and oncologists [104,105]. Collaborative efforts are essential to establish comprehensive clinical guidelines for the management of individuals with lichen sclerosus, ensuring timely interventions and facilitating a proactive stance in mitigating the risk of penile cancer. Therapeutically, the management of lichen sclerosus aims at alleviating symptoms and minimizing the potential for complications, but it also necessitates a broader perspective to address the associated cancer risk. Topical corticosteroids, the mainstay of treatment for lichen sclerosus, can provide relief from symptoms and potentially mitigate inflammation. However, the role of additional therapeutic modalities, such as immune modulators or targeted agents, in altering the natural history of lichen sclerosus and its association with penile cancer remains an evolving area of investigation [106]. Preventively, the recognition of lichen sclerosus as a risk factor for penile cancer underscores the importance of regular surveillance and patient education. Individuals diagnosed with lichen sclerosus should be informed about the potential cancer risk, emphasizing the need for routine clinical examinations and heightened vigilance for any changes in symptoms. Public health campaigns should also focus on raising awareness about lichen sclerosus, its potential association with penile cancer, and the importance of seeking medical attention for timely intervention. In conclusion, lichen sclerosus stands as a complex and significant risk factor for penile cancer, weaving a narrative that intertwines chronic inflammation, clinical challenges, and the potential for malignancy [107]. The elucidation of the molecular underpinnings of lichen sclerosus-associated penile cancer holds promise for advancing our understanding of both conditions and may pave the way for innovative therapeutic strategies.

## 5. Candidate Genes and Pathways

While the specific genes implicated in penile cancer risk are still under exploration, certain candidate genes and pathways have emerged from early investigations. *TP53*, a tumor suppressor gene known for its role in various cancers, has been associated with an increased risk of penile cancer [108,109]. Mutations in *TP53* may disrupt cell cycle regulation and DNA repair mechanisms, contributing to the uncontrolled cell growth characteristic of cancer. Additionally, genes involved in immune response pathways, such as *HLA* (human leukocyte antigen) genes, have been implicated. Variations in *HLA* genes may influence an individual’s ability to mount an effective immune response against infections, including those caused by high-risk HPV subtypes, a well-established risk factor for penile cancer [110,111]. The identification of these candidate genes and pathways marks a significant step forward in understanding the genetic basis of penile cancer. Further research is warranted to elucidate the specific mutations, polymorphisms, and functional consequences associated with these genetic factors. The intricate interplay between genetic factors and high-risk human papillomavirus (HPV) infection adds another layer of complexity to the genetic predisposition of penile cancer. HPV is a well-established risk factor for penile cancer, with subtypes 16 and 18 being the most commonly associated with malignant transformation [112]. Indeed, the expression of the p16INK4a protein was found to be associated with the presence of high-risk oncogenic HPV in penile carcinoma samples [113]. Studies suggest that individuals with specific genetic variations may be more susceptible to persistent HPV infection, increasing their risk of developing penile cancer. The E6 and E7 oncoproteins produced by high-risk HPV subtypes play a pivotal role in promoting cellular transformation and tumorigenesis [114]. Furthermore, DKK1 inhibits canonical Wnt signaling in HPV-positive PeCa cells, and an elevated expression of this protein is linked to higher TNM classification [115]. Genetic factors that influence the host’s immune response to HPV or modulate the cellular response to viral infection may contribute to the progression from HPV infection to penile cancer. Understanding the complex interplay between genetic susceptibility and HPV infection is crucial for tailoring prevention and screening strategies. Individuals with both genetic predisposition and high-risk HPV infection may represent a high-risk subgroup that could benefit from more intensive surveillance and preventive interventions [67,116]. Beyond specific genetic markers, certain hereditary syndromes have been associated with an increased risk of penile cancer [117]. For instance, individuals with a history of lichen sclerosus, a chronic inflammatory skin condition affecting the genital area and often referred to as Balanitis Xerotica Obliterans (BXO), may have an elevated risk [118]. This condition suggests a potential link between chronic inflammation and an increased risk of penile cancer [119]. Furthermore, there is a high overexpression of *NFKB1* mRNA in penile cancer and *NFKB2* mRNA in penile lichen sclerosus, which shows an implication of the *NF-kB* pathway in penile cancer and some dermatoses [118]. Similarly, conditions such as Peutz–Jeghers syndrome, which is characterized by the development of polyps in the gastrointestinal tract and mucocutaneous pigmentation, have been linked to an increased risk of various cancers, including penile cancer [120]. These observations highlight the importance of considering broader genetic syndromes in understanding the genetic landscape of penile cancer. The identification of genetic factors associated with penile cancer risk holds significant implications for clinical practice. Genetic counseling may play a crucial role in informing individuals with a family history of penile cancer about their potential risk. Understanding one’s genetic predisposition allows for personalized risk assessment, screening recommendations, and early detection strategies. Additionally, insights into the genetic basis of penile cancer may pave the way for the development of targeted therapies. Finally, a recent study shows that the abnormal expression of secreted phosphoprotein 1 (SSP1) is closely related to a variety of tumors including penile cancer, so the *SSP1* gene might be an effective biomarker for predicting the prognosis and the efficacy of immunotherapy in PC patients [121]. Precision medicine approaches, tailored to the specific genetic profile of the tumor, hold promise in improving treatment outcomes and reducing the side effects associated with traditional cancer therapies.

## 6. Conclusions

Penile cancer, though rare, presents a significant global health challenge with a profound impact on affected individuals. Despite its low overall incidence, the disease is associated with high mortality and considerable detriment to quality of life. Patients endure not only the typical psychological and emotional toll of cancer but also face unique challenges related to the intimate nature of the disease. The often mutilating treatments can alter body image and masculinity, leading to severe anxiety, depression, and social isolation. The multifactorial etiology of penile cancer underscores the complexity of understanding and addressing the disease. Risk factors such as HPV infection, poor hygiene, smoking, genetic predispositions, and socioeconomic factors all contribute to the development and progression of penile cancer. The global burden of the disease varies significantly by region, with higher incidence rates in South America, Africa, and Southeast Asia compared to developed regions. This disparity highlights the influence of genetic, environmental, and lifestyle factors. Research into the genetic components and socioeconomic determinants of penile cancer is critical for developing targeted prevention and intervention strategies. Advances in genomics and collaborative research efforts have begun to elucidate potential genetic markers, while the exploration of socioeconomic factors reveals significant health inequities. By comprehensively analyzing these risk factors, it would be possible to enhance prevention strategies, improve early detection, and develop more effective treatments. Understanding penile cancer’s epidemiology and risk factors is essential for tailoring public health initiatives and clinical practices. By addressing the multifaceted nature of the disease, it is possible to work towards reducing its incidence and improving patient outcomes, ultimately alleviating the burden on individuals and society. Enhanced awareness, combined with targeted research and interventions, holds the promise of better management and prevention of this challenging condition.

## Figures and Tables

**Table 1:** Histopathological classification of penile cancer according to WHO (2022).

Histology	Features
**HPV-independent SCC**	
**Usual**	Most common, various degrees of differentiation. Include the pseudohyperplastic and the pseudoglandular variants.
**Verrucous Carcinoma**	Well differentiated, broad-based. Relatively indolent.
**Cuniculatum**	Endophytic labyrinthine growth pattern.
**Papillary**	Papillae covered by well- to moderately differentiated cells.
**Sarcomatoid**	Most aggressive and worse prognosis.
**Mixed**	Two or more subtypes in the same specimen.
**HPV-associated**	
**Basaloid**	Uniform basaloid cells in nests or sheets with comedonecrosis or keratinization.
**Warty**	Condylomatous papillae with central fibrovascular cores.
**Clear cell**	Nests or sheets of cells with ample, clear cytoplasm with central or geographical necrosis.
**Lymphoepithelioma-like**	Poorly differentiated cells intermixed with dense lymphoplasmacytic and eosinophilic infiltrate.
**Mixed**	Mainly warty–basaloid carcinoma.
**Others**	
**SCC not otherwise specified**	Keratinizing carcinoma. Used when evaluation of p16 is not available.
**Adenosquamous**	Squamous tumor nests intermixed with a minor mucinous glandular component.
**Mucoepidermoid**	No clear separation from the adenosquamous.

## Data Availability

No new data were created or analysed in this study. Data sharing is not applicable to this article.
